# A meta-analysis of the effects of crop residue return on crop yields and water use efficiency

**DOI:** 10.1371/journal.pone.0231740

**Published:** 2020-04-27

**Authors:** Xingli Lu

**Affiliations:** College of Agronomy, Ningxia University, Yinchuan, Ningxia, China; Tennessee State University, UNITED STATES

## Abstract

After harvesting agricultural crops, the residue can be returned to the soil as mulch. This study performed a meta-analysis of previous research to investigate the effects of crop residue return and other factors on crop yields and water use efficiency (WUE). Overall, the results show that crop residue return increases crop yields by 5.0% relative to crops grown without it. The greatest increases in yield for crops grown with returned residue were associated with average annual temperatures < 10 °C (yield increase = 7.6%), rainfall ≥ 800 mm (9.5%), plowing depth ≥ 20 cm (6.5%), corn crops (8.0%), growth of a single crop per year (10.1%), no irrigation (11.9%), nitrogen (N), and potassium (K) fertilization (20.0%), and low nitrogen application rates of 0–100 kg N ha^-1^ (10.8%). The effects of crop residue return on crop yields were found to vary according to the following soil properties: organic matter content ≥ 15 g kg^-1^ (yield increase = 9.4%), available nitrogen content ≥ 100 mg kg^-1^ (10.3%), and pH ≤ 6.5 (11.2%). The greatest magnitudes of increase in WUE associated with crop residue return were associated with corn (yield increase = 13.7%), medium nitrogen content (100–150 kg ha^-1^; 23.3%), high soil organic matter (≥ 15 g kg^-1^; 25.5%) and low air temperatures (< 10 °C; 19.9%). In addition, our results suggest that crop residue return might be most effective in increasing crop yields and WUE in corn crops, crops with a tillage depth ≥ 20 cm, crops grown with moderate nitrogen fertilization (0–150 kg ha^-1^), growth of a single crop per year, high soil organic matter content (≥ 15 g kg^-1^), and cold conditions (< 10 °C). Overall, the results of this meta-analysis suggest that crop residue return can increase crop yields and WUE, with the relationship being mainly affected by climatic conditions, plowing depth, fertilization management, crop types, and soil properties.

## Introduction

The production of residues associated with 27 food crops has been evaluated at 3758 × 10^6^ Mg yr^-1^ [1]. Returning crop residue to the soil can avoid the greenhouse gas emissions caused by burning it [2] while improving the soil organic matter content, soil physical properties, water use efficiency (WUE), soil structural stability, soil expansion, and capacity expansion, as well as reducing soil bulk density [3, 4, 5]. Moreover, crop residue return can increase crop yields and quality [6, 7]. Thus, crop residue return plays important roles in sustainable agriculture and environmental protection.

However, some studies have shown that crop residue return can have negative effects on the environment and crop yields [8, 9]. For example, the decomposition of crop residues consumes soil-available nitrogen, which is not conducive to crop growth and yields [10]. Reductions in crop yields induced by crop residue return are caused by imbalances in the soil carbon:nitrogen ratio [11]. In other cases where crop residue return does not increase crop yields, the emergence rate is poor because of improper farming methods or poor-quality seeding [12]. Crop residue return improves the soil’s water retention capacity, which increases wheat growth during the nutritional growth period and reduces the harvest index of wheat [13]. Zhou et al. [14] showed that crop residue return decreases ground temperature and delays crop growth, which reduces yields. Thus, although there has been extensive research on the effects of crop residue return, the results are inconsistent or contradictory because of differences in soil conditions, planting systems, and climatic conditions [15, 16].

Meta-analysis has been used to synthesize information from diverse studies performed under various conditions. It can provide effect sizes, which are calculated as the response of a treatment relative to that of an untreated control [17, 18]. This can solve problems that can’t be solved by a single study. The effects of crop residue return on crop yields and WUE have been widely investigated using different methods, including meta-analysis [19, 20, 21]. For example, by applying a meta-analysis method, Yu et al. [20] showed that crop residue return can significantly improve maize grain yields and WUE in Northern China because it enhances the soil hydrothermal environment. Crop residue return can clearly increase crop yields and WUE, but the magnitude of the effect may vary according to the site and agronomic management regime [22, 23]. However, the understanding of the effects of crop residue return on crop yields and WUE under different types of agricultural management (e.g., different crop types, irrigation conditions, tillage type, fertilizer conditions, and cropping system type), experimental durations, climatic conditions, and soil properties (such as soil organic matter and pH) remains incomplete. Therefore, we conducted a meta-analysis to evaluate the impacts of crop residue return on crop yields and WUE relative to those of crops grown without crop residue treatment (referred to here as *no-straw*). These effects were also studied in relation to agricultural management strategies (crop type, tillage type, fertilizer type, depth of tillage, amount of N fertilizer, cropping system type), climatic conditions (mean annual temperature, precipitation), soil properties, and experimental duration.

## Materials and methods

### Data sources

Experimental research papers were identified via a search for field-based reports on the effects of crop residue return on crop yields and WUE that were published prior to 2018. Chinese and English databases were used, including the China Knowledge Network, Weipu, Wanfang, Web of Science, Springer, Engineering Village, and Google Scholar. The keywords included “straw”, “mulching”, “residue”, and “yield”. To reduce uncertainties and meet the requirements of the meta-analysis, the studies were selected using the following criteria: (1) the experiment must have been conducted in the field; (2) the experimental duration must have been ≥ 2 years; (3) there were ≥ 3 replicates; (4) the test site and year were clearly identified; (5) the test treatments included both crop residue return and no return; and (6) in cases where many years of production data were included, only the most recent year was used. If standard deviations (SDs) were given in an original paper, they were used directly. If standard errors (SEs) were given, they were converted to SDs using Eq (1):

SE=SDn (1)

**Fig 1:**
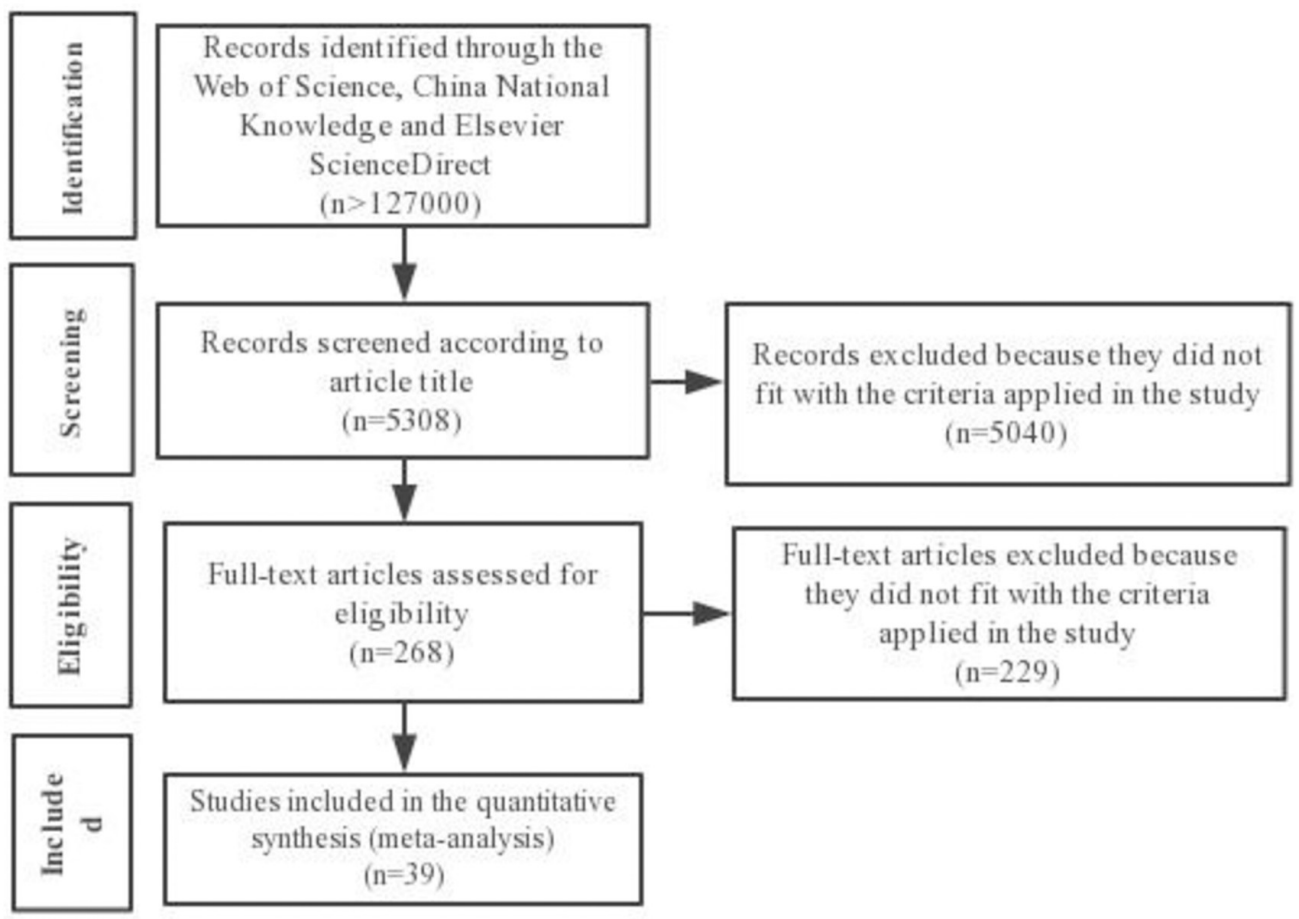
Flowchart diagram of the process applied to obtain the literature data to build a database for the study.

In other cases, the average coefficient of variation through the whole dataset was used to calculate the standard deviation according to the method of van Groenigen et al. [24]. A total of 39 articles were selected after screening 268 papers (Fig 1 and S1 Table).

The WUE was calculated according to Li et al. [2]:

$$WUE=\frac{Yield}{ET_{a}}$$ (2)

where *Yield* is the grain yield (kg ha^−1^) and *ET*_*a*_ is actual crop evapotranspiration (mm).

The type of crops included in the database search comprised oilseed rape (*Brassica napus* L.), corn (*Zea mays* L.), soybean (*Glycine max Merri* L.), wheat (*Triticum aestivum* L.), rice (*Oryza*.*sativa* L.), pea *(Pisum sativum* L.), and cotton (*Gossypium spp*). Irrigation conditions were separated into three categories: no irrigation, irrigation, and paddy. We grouped irrigation water types into two groups: brackish water and fresh water. The tillage types were classified into five groups: rotary tillage, chisel plow tillage, no-tillage, harrow plowing tillage, and moldboard plowing tillage. The fertilizer types were partitioned into seven groups: no fertilizer, nitrogen (N), phosphorus (P), and potassium (K) + organic fertilizer, NPK, NP + organic fertilizer, NP, NK, and N. The depth of tillage was classified into two groups: ≥ 20 cm and < 20 cm. The rate of N fertilizer application was divided into the following classes: > 150 kg ha^-1^, 100–150 kg ha^-1^, 0–100 kg ha^-1^ and 0 kg ha^-1^. The experimental durations were divided into three categories: ≥ 10 years, 3–10 years, and ≤ 2 years. The cropping system types were divided into two groups: one crop per year and two crops per year. The soil organic matter was divided into three groups: > 15 g kg^-1^, 10–15 g kg^-1^, and ≤ 10 g kg^-1^. The soil-available nitrogen content was divided into two categories: < 100 mg kg^-1^ and ≥100 mg kg^-1^. The soil pH value was divided into three categories: > 8.0, 6.5–8.0, and ≤ 6.5. The mean annual temperature was divided into three categories: > 15 °C, 10–15 °C, and < 10 °C. Precipitation was divided into three categories: ≥ 800 mm, 400–800 mm, and < 400 mm. The soil total nitrogen content was classified into two categories: < 1 g kg^-1^ and ≥ 1 g kg^-1^ (Table 1).

**Table 1:** Categorical variables (*Var*), total number of paired observations of crop yield for crop residue return and no-straw treatments (k), specific levels of each Var (L), between-group heterogeneity (Qb), and significant P values produced by the meta-analysis.

No	Var.	k	L1	L2	L3	L4	L5	L6	L7	Qb	*p*
1	Crop types	144	Rape	Corn	Soybean	Wheat	Rice	Pea		21.5166	0.0094
2	Irrigation condition	119	No irrigation	Irrigation	Paddy					12.5712	0.0064
3	Irrigation water types	89	Brackish water	Freshwater						23.3343	0.0008
4	Tillage types	108	Rotary tillage	Chisel plow tillage	No tillage	Harrow ploughing tillage	Mouldboard ploughing tillage			12.7747	0.0272
5	Fertilizer types	142	No fertilizer	NPK+organic fertilizer	NPK	NP+organic fertilizer	NP	NK	N	31.6973	0.001
6	Depth of tillage (cm)	77	≥20	<20						5.3109	0.0418
7	Amount of N fertilizer (kg ha^-1^)	131	>150	100–150	0–100	0				11.57	0.021
8	Experimental duration (year)	145	≥10	3–10	≤2					9.5218	0.0252
9	Cropping system types	134	One crop a year	Two crops a year						12.0566	0.0028
10	Soil organic matter (g kg^-1^)	109	>15	10–15	≤10					9.0164	0.0322
11	Soil-available nitrogen content (mg kg^-1^)	52	<100	≥100						8.3198	0.008
12	Soil pH value	68	>8	6.5–8.0	≤6.5					21.0101	0.0006
13	Mean annual temperature (°C)	54	>15	10–15	<10					4.3849	0.1420
14	Precipitation (mm)	102	≥800	400–800	<400					11.0903	0.016
15	Soil total nitrogen content (g kg^-1^)	79	<1	≥1						2.8911	0.1386

### Calculation of size of the effects

The effect sizes were calculated using the yield, WUE, standard deviation (SD), and number of replicates used in each study [25].

ln(R)=ln(Xex/Xck), (3)

where R is the effect size, and Xex and Xck are the yields and WUEs for the crop residue return and no-straw treatments, respectively.

The variance (Var) was calculated after Xu et al. [26]:

$$v=\frac{SD_{ex}^{2}}{n_{ex}\times X_{ex}^{2}}+\frac{SD_{ck}^{2}}{n_{ck}\times X_{ck}^{2}}$$ (4)

where *SD*_*ex*_ and *SD*_*ck*_ represent the SDs of the yield and WUE for crop residue return and no-straw treatments, respectively; and *n*_*ex*_ and *n*_*ck*_ are the sample sizes for these treatments, respectively.

The weight (*w*) of each effect size was calculated as follows:

w=1v (5)

We calculated the mean effect size as:

$$\bar{\text{ln}(R)}=\frac{\sum \text{ln}\;R_{i}\times w_{i}}{\sum w_{i}}$$ (6)

Where ln *R*_*i*_ and *w*_*i*_ are the effect sizes and weights of the corresponding observations, respectively. Thus, the 95% confidence interval (CI) of ln(R) was calculated as:

$$95\%CI=\bar{\text{ln}(R)}\pm 1.96SE_{\bar{\text{ln}(R)}}$$ (7)

where $SE_{\bar{\text{ln}(R)}}$ is the SE of lnR and was calculated as:

$$SE_{\bar{\text{ln}(R)}}=\sqrt{\frac{1}{\sum w_{i}}}$$ (8)

To reflect the effect of crop residue return on yield more intuitively, ln(R) was converted to Y, the magnitude of the increase in yield, and WUE [25]:

Y(%)=(Exp(lnR)−1)×100% (9)

If the upper and lower limits of the 95% confidence interval for Y were both greater than zero, then it was concluded that crop residue return improved crop yield or WUE compared to no-straw treatment. If both limits were < zero, then crop residue return was considered to have decreased crop yield or WUE. If the 95% CI included zero, then it was concluded that there was no difference in crop yield or WUE between the crop residue return and no-straw treatments.

### Statistical analysis

Meta-analysis was performed using Metawin 2.1 software [27, 28]. The mean effect sizes were evaluated with a random-effects model. The 95% CI for each mean effect size was estimated by applying bootstrapping with 4999 iterations [27]. Mean effect sizes were considered significantly different when the 95% CIs did not overlap, and the Gaussian distribution lines were made using Origin 8.5 software. The images were processed using GraphPad Prim 6.0 software.

## Results

### Test of publication bias

**Fig 2:**
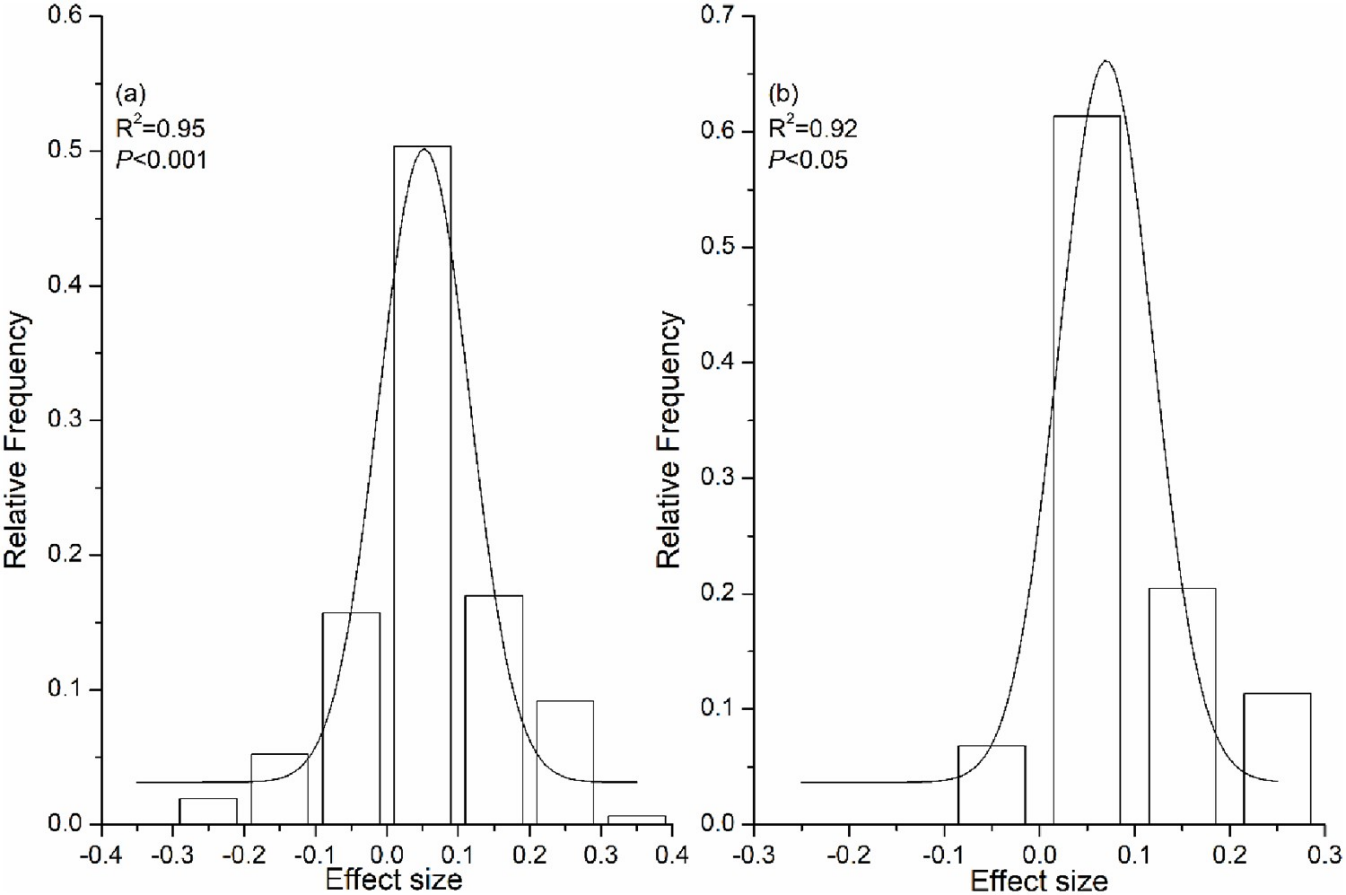
Frequency distribution of effect sizes for yield (a), and WUE (b) responding to crop residue return compared to no-straw. Solid lines are fitted normal (Gaussian) distributions of frequency data sets.

Our dataset consisted of 193 comparisons of crop residue return versus no-straw treatments, comprising 146 comparisons of yield and 47 of WUE. These comparisons were taken from 39 studies, 13 of which were published in English and 26 in Chinese. The frequency distributions of the effect size were normal Gaussian distributions for the yield and WUE, suggesting that the datasets were homogeneous (Fig 2 and S2 Table; [29]).

### Impact of crop types and irrigation conditions

**Fig 3:**
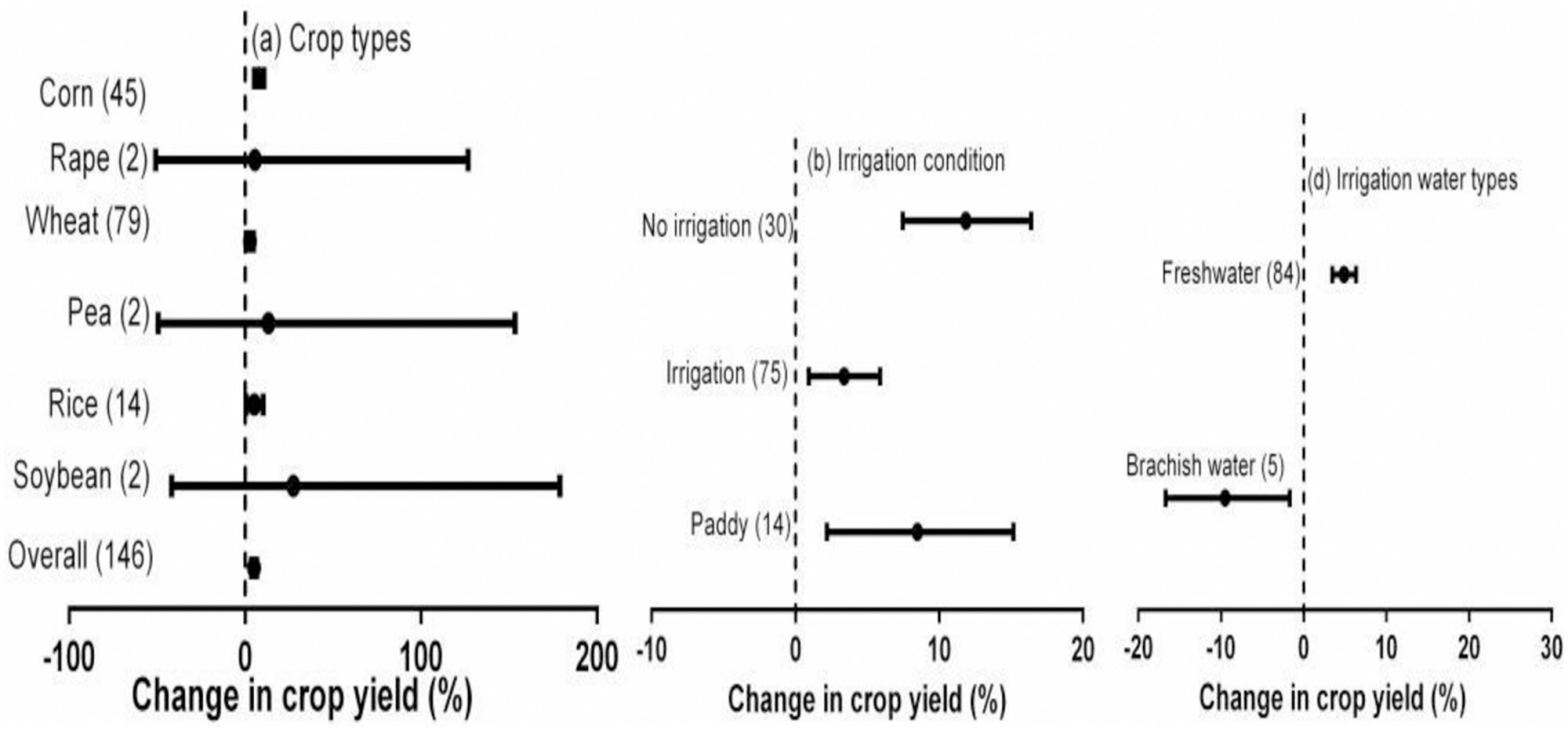
Effect of crop residue return on crop yield for: (a) different crop types; (b) irrigation conditions; (c) irrigation water type. Error bars represent the mean value ± 95% CI.

The impact of crop residue return on crop yield was significantly affected by crop type (*P* < 0.05; Table 1, Fig 3a). The results for soybean, rape, and pea overlapped zero, suggesting no significant effect of crop residue return on their yields. Wheat crops had the smallest increase in yield associated with crop residue return (2.7%). The effects of crop residue return on the yields of rice and corn crops were 5.3% and 8.0%, respectively (Fig 3a).

The effect of crop residue return on crop yield was also impacted by the irrigation strategy (Table 1, Fig 3b). Without irrigation, crop residue return increased crop yield by 11.9% compared to no-straw. Crop residue return increased crop yields by 8.5% and 3.4% for paddy and irrigated crops, respectively. The type of irrigation water used also significantly affected the effect of crop residue return on crop yield (Table 1, Fig 3c). Crop residue return increased the yields of crops irrigated with fresh water by 4.9%, whereas those of crops irrigated with brackish water were reduced by 9.5% compared to no-straw.

### Impacts of tillage and fertilizer conditions

**Fig 4:**
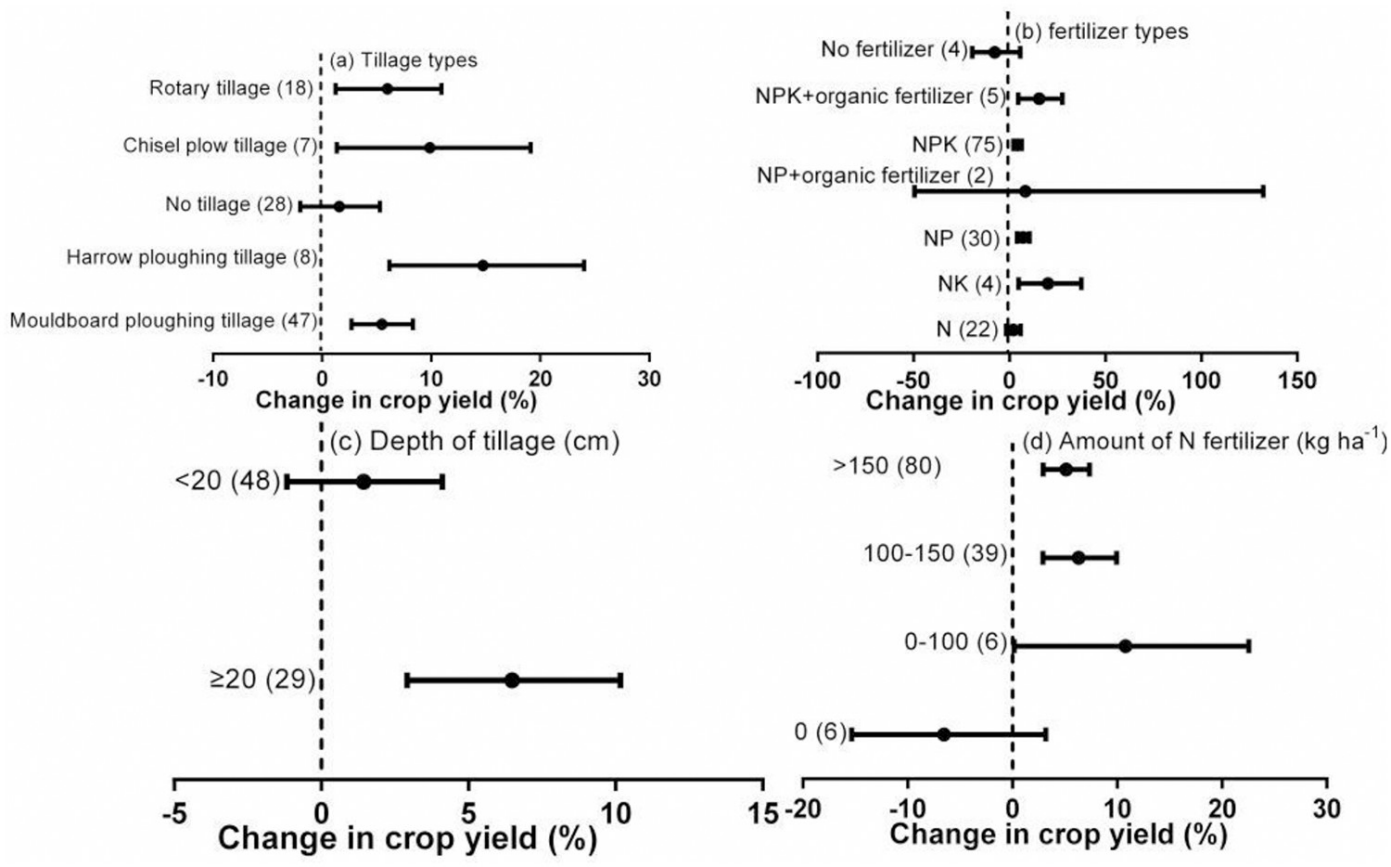
Effect of crop residue return on crop yield under different tillage types (a), depth of tillage (b), fertilizer types (c), and amount of N fertilizer (d). Error bars represent the mean value ± 95% CI.

The effect of crop residue return on crop yield varied with the type of farming system (Table 1, Fig 4). For the different tillage measures, the order of yield increase associated with crop residue return was: harrow plowing tillage (14.7%) > chisel plow tillage (9.9%) > rotary tillage (6.0%) > mouldboard plowing tillage (5.5%). No difference in crop yield was recorded between crop residue return and no-straw treatments under no-tillage.

The type of fertilizer also significantly affected the impact of crop residue return on crop yield. Where no fertilizer was used, crop residue return had no effect on crop yield compared with no-straw, but increases in crop yield were observed when crop residue return was used in conjunction with fertilization by NK (20.0%), NP (7.2%), NPK (4.3%), and NPK+ organic fertilizer (15.5%; Fig 4b).

Crop residue return increased yields by 6.5% for plow depths ≥ 20 cm (Table 1, Fig 4c) but no difference was recorded for depths < 20 cm. The amount of N fertilizer also significantly affected the impact of crop residue return on yield (Table 1, Fig 4d). Without N fertilizer, crop residue return had no impact on yield compared with no-straw, but at low rates of N application (0–100 kg N ha^-1^), crop residue return increased crop yield by 10.8%, which was the highest level of improvement with the various rates of N fertilization. At medium (100–150 kg N ha^-1^) and high (>150 kg N ha^-1^) rates, crop residue return increased yields by 6.3% and 5.1%, respectively, compared to no-straw.

### Impact of experimental duration, cropping system type, and climatic conditions

**Fig 5:**
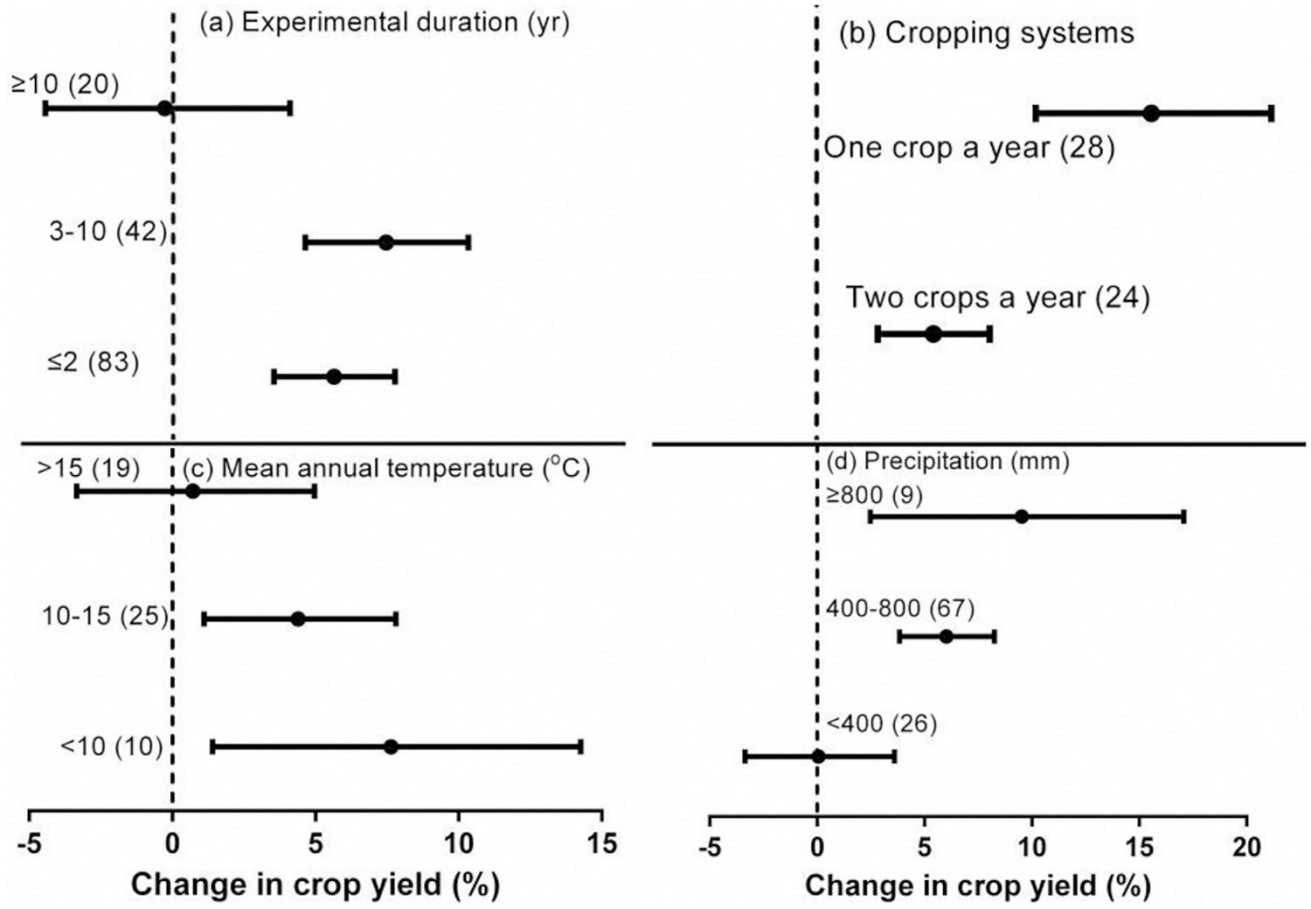
Effect of crop residue return on crop yield under different experimental duration (a) and cropping system types (b), mean annual temperature (c), and precipitation (d). Error bars represent the mean value ± 95% CI.

The number of years over which crop residue return was practiced significantly affected the effect of crop residue return on crop yield (Table 1, Fig 5a). The greatest increases in crop yield were observed with experimental duration of crop residue return of 3–10 years (7.1%). No difference in crop yield was observed between treatments with experimental duration > 10 years. An increase in yield of 5.4%, compared to no-straw, was observed for experiments where crop residue return occurred for ≤ 2 years (Table 1, Fig 5a).

The type of cropping system also significantly affected the yield increase associated with crop residue return. The increase was 10.1% in cases with one crop per year and significantly higher for cases with two crops a year (3.5%; Table 1, Fig 5b).

Although the between-group difference was not significant, crop residue return did increase yield in cooler areas (Table 1, Fig 5c). Crop residue return increased yields by 9.5% and 6.0% with annual rainfall of ≥ 800 mm and 400–800 mm, respectively, compared to no-straw. There was no difference when annual rainfall was < 400 mm (Table 1, Fig 5d).

### Impact of soil properties

**Fig 6:**
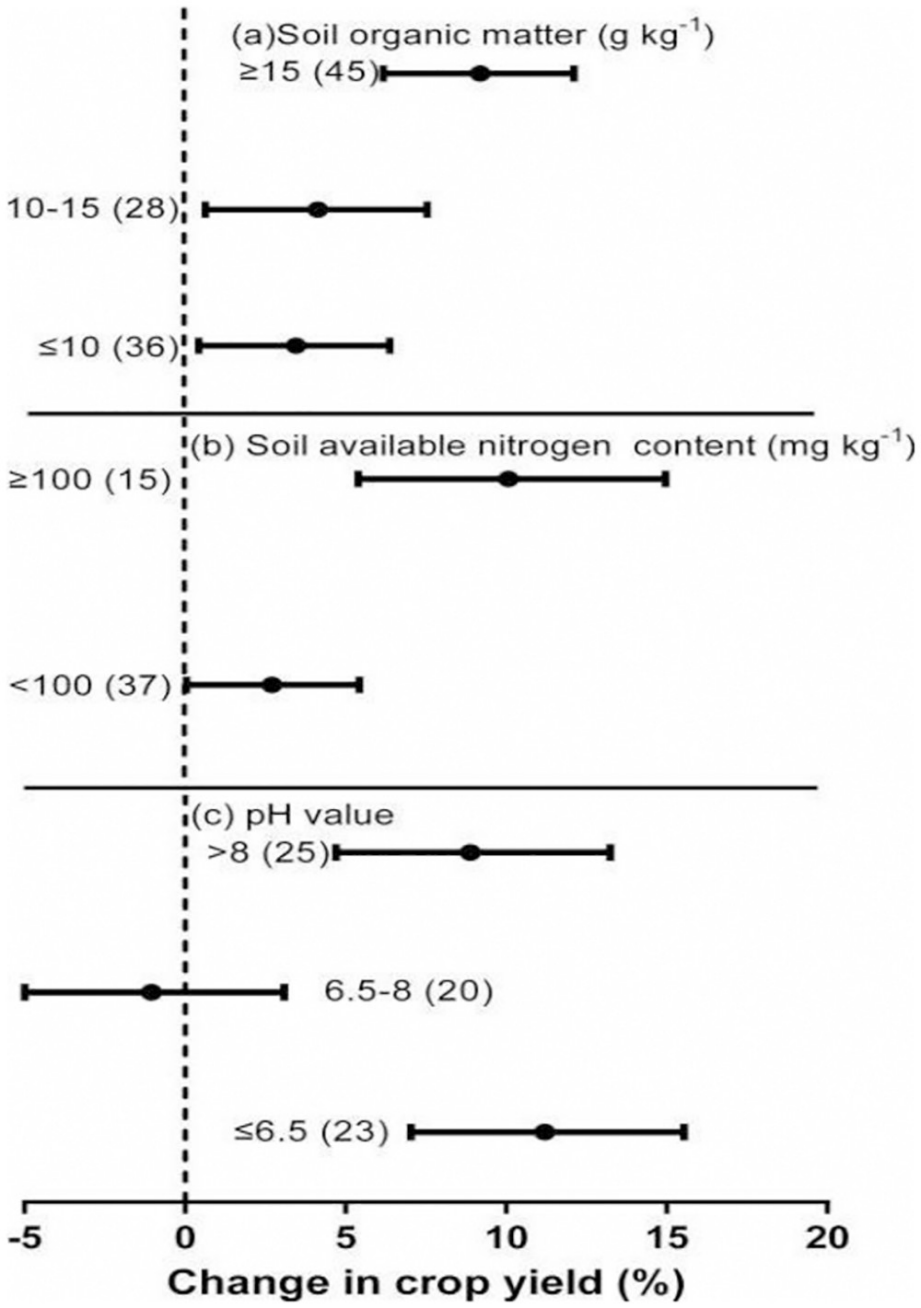
Effect of crop residue return on crop yield under different soil properties (i.e., a, soil organic matter; b, soil-available nitrogen content; c, soil pH value). Error bars represent the mean value ± 95% CI.

Soil properties, including soil organic matter, soil nitrogen content, and pH, had a significant impact on the effect of crop residue return on crop yield (*P* < 0.05; Table 1, Fig 6). The increase in yield associated with crop residue return was greatest in cases where the soil organic matter content was > 15 g kg^-1^ (9.4%) and the least (3.5%) when it was low (≤10 g kg^-1^). Crop residue return increased yield by 4.2% when the soil organic matter content was 10–15 g kg^-1^.

The soil-available nitrogen content also significantly affected the increase in crop yield induced by crop residue return (*P* < 0.05; Table 1). The higher increase in yield was observed when the soil-available nitrogen content was ≥ 100 mg kg^-1^ (10.3%) and the lowest increase in yield (2.8%) was observed when the soil-available nitrogen content was < 100 mg kg^-1^. The yield increase caused by crop residue return was not affected by the total nitrogen content of different soils (Table 1). In contrast, the soil pH significantly affected the yield increase caused by crop residue return (Table 1, Fig 6c). At pHs of 6.5–8, crop residue return had no effect on yield compared with no-straw treatment, while at pH ≤ 6.5 it increased yield by 11.2% and at pH > 8 it increased yield by 8.9%.

### Effect size of WUE and its influences

**Fig 7:**
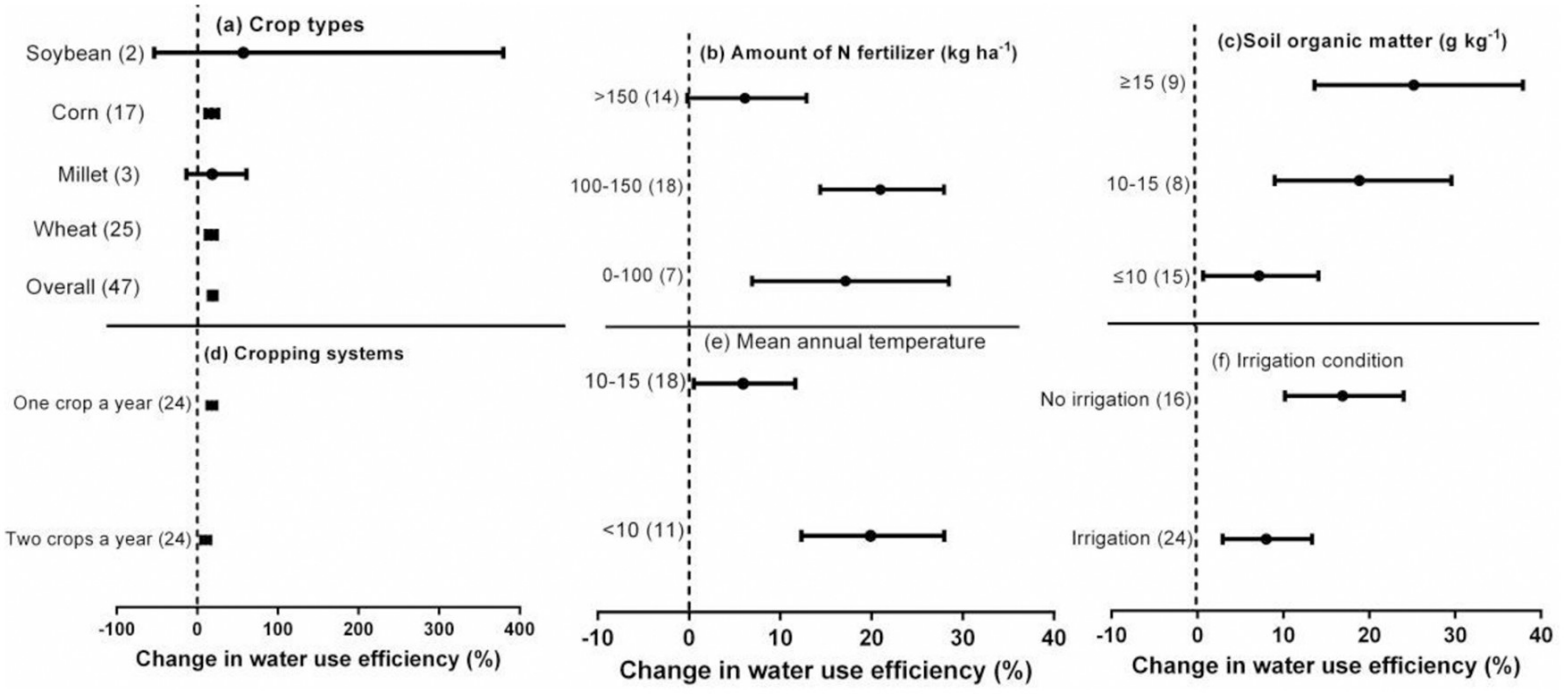
Effect of crop residue return on WUE under different crop types (a), amount of N fertilizer (b), soil organic matter (c), cropping systems (d), mean annual temperature (e), and irrigation condition (f). Error bars represent the mean value ± 95% CI.

Crop residue return significantly increased WUE for corn (13.7%), and wheat (13.2%) compared to no-straw (*P* < 0.05; S3 Table, Fig 7a). In contrast, no difference in WUE was recorded between crop residue return and no-straw treatments for the different tillage types, fertilizer types, experimental durations, or depths of tillage (S3 Table). The effect of crop residue return on WUE was significantly affected by the amount of N fertilizer, soil organic matter, cropping system, mean annual temperature, and irrigation conditions (S3 Table, Fig 7).

The greatest increase in WUE caused by crop residue return was recorded for crops with medium rates of N fertilizer application (100–150 kg ha^-1^), with a mean increase of 23.3%. Increases in WUE caused by crop residue return were 25.5% for ≥ 15 g kg^-1^, 19.2% for 10–15 g kg^-1^, and 7.5% for ≤ 10 g kg^-1^ soil organic matter. The increase in WUE induced by crop residue return was greater at temperatures < 10 °C (19.9%) than at 10–15 °C (5.9%; S3 Table, Fig 7e).

## Discussion

### Effect size of crop residue return on crop yield

In this study, the impacts of crop residue return on crop yield and WUE were investigated using a meta-analysis of studies from China and abroad that considered the effects of different climatic conditions, tillage practices (tillage type and depth), crop types, cropping systems, irrigation conditions, fertilizer application strategy (fertilizer types and amounts of N fertilizer application), soil properties (soil organic matter, soil pH, and soil-available nitrogen content). The results show that crop residue return significantly increased the average crop yield by 5.0% compared to no-straw treatment. The main reasons for the increase in crop yield caused by crop residue return are: (i) it can increase soil porosity, reduce soil compaction and bulk density [29], improve soil aeration and water status, and reduce water consumption [30]; (ii) it increases the contents of organic matter, available nutrients, fulvic acid, and humic acid in the soil, promotes the release of slow-acting potassium in the soil [31, 32], reduces the amount of chemical fertilizers required [33], improves the soil environment [34], increases the leaf area of plants, and promotes the transport of photosynthetic substances to the grain [35], thus improving crop quality [36]; and (iii) crop residue is rich in organic matter, which can provide abundant carbon sources for microorganisms in the soil, stimulate microbial activity, improve soil fertility [37], promote the reproduction of earthworms [38], and increase the diversity of soil arbuscular mycorrhizal fungi [39], ultimately increasing crop yield.

### Factors affecting the effect size on crop yield

#### Crop types and irrigation conditions

The yield-increasing effects of crop residue return differ according to the crop. Zhang [40] found that the return of wheat crop residue increased the yield of soybeans more than that of wheat. Wang et al. [41] showed that crop residue return increased soil moisture, lowered soil temperature, and provided cooling effects during the early stages of soybean growth, which ultimately increased the soybean yield. Ji et al. [42] reported that the magnitude of crop residue return on wheat yield was greater than that of corn because of the soil environment before planting and the climatic conditions during growth. Zhang et al. [43] also showed that crop residue return can significantly increase wheat yield but has no effect on rice yield due to the different cultivation and climate conditions. Our results indicate that the impact of crop residue return on crop yield is complex and variable.

With respect to irrigation, the results of the meta-analysis showed that increases in crop yield due to residue return were greatest without irrigation. This might be because water is the main factor limiting crop yields in drought conditions. The water type also affects the effect of crop residue return on crop yield. A negative effect of crop residue return on crop yield was observed under brackish irrigation. Similarly, Levy [44] and Zheng et al. [45] reported that brackish water can decrease crop growth and yield, as salt damage makes it difficult for plant roots to absorb water. However, Lu et al. [46] showed that applying brackish water increased corn yield and WUE under high crop residue return conditions. Therefore, long-term studies regarding the impacts of crop residue on crop yields under brackish irrigation are needed to identify effective methods of such irrigation.

#### Tillage, fertilizer treatments, and soil properties

Soil with good physical properties can promote the production of dry matter and crop yields. The results of our study showed that the magnitude of increase in crop yields due to crop residue return varied according to the tillage methods (harrow plowing, chisel plow tillage, rotary tillage, and no tillage) and ploughing depths. The larger magnitude of increasing in crop yield was recorded for harrow plowing tillage, and chisel plow tillage in related to other tillage treatments. Similarly, Other studies showed that the main reasons proposed for the increased yield are that chisel plow tillage is conducive to the formation of a crop-favoring structure in the ploughed layer, promotes soil water infiltration, reduces soil bulk density, enhances rooting in the ploughing layer, enhances soil moisture movement, and promotes the absorption of nutrients and water by crop roots [47, 48, 49, 50, 51]. The results of Huang et al. [52] and Xu et al. [7] show that chisel plow tillage in conjunction with crop residue return can improve crop yields by promoting the accumulation and transfer of dry matter.

In addition, our results showed that no difference in crop yield was recorded between crop residue return and no-straw treatments under no tillage. Our results are consistent with Huang et al. [53]. However, some studies showed that crop residue return combined with no-tillage methods decreased crop production, because soil temperatures were cooler, which reduced emergence and crop growth, and increased soil compaction and micro-nutrient deficiencies [54, 55]. Yadvinder-Singh et al. [56] also reported that the decomposition rates under no tillage was lower than that of tillage treatment because of the reduction in residue-soil contact, which ultimately reduced crop yield. In contrast, Zhou et al. [57] reported that crop residue return could increase crop yield under no tillage mainly due to the improvement of soil fertility. These differences in the effects of crop residue return under different tillage treatment on crop yield suggest that further work is necessary to determine its impact on crop yields with different soil tillage types and depths.

The most important factor that affects crop yield is the type and application rate of chemical fertilizer. The results of this study show that the increases in yield associated with crop residue return ranged from 2.0% to 20.0% for different fertilizer types. Huang et al. [58] showed that crop residue return combined with nitrogen fertilization increased crop yield while, in contrast, yield was reduced when crop residue was returned without nitrogen fertilizer. The combination of nitrogen fertilizer and crop residue return has been reported to significantly improve soil fertility and increase winter wheat yield by 7.5% [59]. The increase in crop yield after a single cycle of crop residue return is not obvious, which might be related to subsequent changes in soil hydrothermal conditions [60]. It was noted by Rathke et al. [61] that a supply of soil nitrogen associated with crop residue return is the main factor limiting increases in crop yield. Zhou et al. [57] also showed that crop residue return plus nitrogen fertilization can increase the nitrogen-use efficiency and soil nitrogen content as nitrate, which indirectly improves crop yield. In addition, our results showed that crop residue return is more beneficial with low amounts of N (0–150 kg ha^-1^) than high amounts (>150 kg ha^-1^). Similarly, previous meta-analyses regarding the effect of mulching on potato yields showed that the mean impacts of crop residue return on potato yields were greater with low amounts of N than high amounts [19]. Therefore, our results indicated that crop residue return was a recommended measure to reduce the requirement of chemical fertilizer, which was benefit for the environment sustainable development.

Moreover, our results indicate that the magnitude of increase in crop yield was greater in highly fertile soil (higher soil organic matter and soil-available nitrogen content) than in low-fertility soil. Similarly, Limon-Ortega et al. [62] found that when soil fertility was low or when the application of N fertilizer was unreasonable, crop residue return did not help crop growth. In fact, it can have adverse effects and even lead to reduced yields; however, when soil fertility is high or when N fertilizer is applied properly, crop residue return promotes crop growth and increases yield. Tang et al. [63] also reported that crop residue return increased crop yield by improving soil fertility on the Chengdu Plain of China. Crop residue return can improve crop yields by increasing the use-efficiency of elements (e.g. N, P) via improvement in soil water utilization, and promote the absorption of elements (e.g. N, P) by crops and the microbial community [64]. In addition, the magnitude of increasing in crop yield under crop residue return was larger when the pH value ≤6.5, which indicated that crop residue return might be benefit for the improvement of the acid soil. Our results suggest that appropriate consideration of soil fertility and fertilizer application probably increases crop yields.

### Experimental duration and climate conditions

The effect of crop residue return on crop yields was affected by the duration of the experiments conducted in previous studies. The results show that crop residue return caused the highest increases in crop yields in experiments conducted over 3–10 years. No difference in crop yield was observed between crop residue return and no-straw treatments in experiments conducted for > 10 years. Similarly, Zhao et al. [21] reported that crop residue return only increases crop yields over the short-term (< 10 years) because the temporal dynamics of yield are more sensitive to climatic conditions than to crop residue return itself. However, Xu et al. [7] showed that crop residue return increased crop yields in winter wheat and summer maize after 11 years mainly due to the enhancement of soil organic carbon storage. Meanwhile, Song et al. [65] showed that, compared with conventional tillage treatment, no-tillage with crop residue return reduced rice and wheat yields by the third year, mainly because of a reduction in seedling growth induced by using large amounts of straw at the beginning two years. Thus, the impact of crop residue return on crop yield depended on the duration of the experiment. Furthermore, our results suggest that yield response to crop residue return is quite variable and the normal amount of crop residue that is returned to the field may sometimes need to be reduced to increase crop productivity and the efficiency of the practice. However, this topic needs further investigation.

The greatest magnitude of increase in crop yield was recorded with rainfall of ≥ 800 mm. In contrast, Wang et al. [66] reported that crop residue return could significantly increased wheat yield and rainfall storage as compared with no straw during the dry years, while it did not increase wheat yield during years with >500 mm annual rainfall. Wicks et al. [67] noted that crop residue return reduced wheat production in wet soil under rainy and cold climate conditions. Crop residue return could reduce crop yield because of the reduction of soil temperature in cool climates compared to no straw [68]. These differences might be due to the reason: in the present study, the experiments with rainfall ≥800 mm were conducted in subtropical monsoon climate, which might be benefit for the decomposition of crop residue rapidly [57, 69]. Previous study also showed that alternating dry and wet conditions promote cycling between aerobic and anaerobic conditions, ultimately increased microbial diversity in the soils and the decomposition of soil organic matter [70]. The results of our study indicate that the effect of crop residue return on crop yield is affected by many factors such as rainfall. However, more crop residue return studies regarding crop yield under different climate conditions are needed to draw more representative conclusions.

### WUE and its influences

Our results show that crop residue return significantly increases WUE by 14.8%, with pronounced increases in WUE for corn and wheat crops. These results are consistent with those of Chakraborty et al. [71], who showed that crop residue return enhanced wheat WUE in India by 13%–25% compared to no-straw treatment. Similarly, crop residue return was reported to conserve soil moisture and reduce the daily variation in soil temperature [64, 72], which ultimately increase WUE. Crop residue return can improve WUE by enhancing crop yield, conserving soil water storage, and reducing soil water losses during the whole growth period under normal soil water and even slight drought conditions; however, it can also reduce WUE, mainly by reducing crop yields due to competition with microorganisms for soil water under drought conditions [73, 74].

In addition, the largest increases in WUE caused by crop residue return were recorded with medium amounts of N fertilization, growth of one crop per year, high soil organic matter content (≥ 15 g kg^-1^), low mean annual temperature (< 10 °C), and irrigation. Therefore, a higher crop yield might also contribute to enhanced WUE under the conditions considered in the present study. Similarly, Li et al. [19] showed that the effects of residue return on potato WUE varied according to the soil’s basic fertility, air temperature, and inorganic fertilizer conditions. Thus, our results also indicate that the effect of crop residue return on WUE is affected by many factors such as the amount of N fertilization, soil organic matter, and mean annual temperature.

### Limitations of the study

Increased yields due to crop residue return can contribute to food security. Because of the limitations in the available data, this study only focused on yields at a few sites. The effects of crop residue return on crop yields at different sites are expected to vary, which should not be ignored. Thus, crop residue return needs to be analyzed at different spatial scales to better identify its effects on crop yields and WUE. In addition, the data used in the present study were only taken from studies that satisfied the five inclusion criteria, which may not represent all relevant studies. A lack of certain meta-data (e.g., water types, fertilization methods, residue management, previous crops, and soil properties) made it difficult to include those studies’ results in this meta-analysis. Thus, we recommend that more detailed and standardized research regarding the effects of crop residue return on crop yields under various environmental and management conditions is conducted to obtain more comprehensive conclusions.

## Conclusions

The meta-analysis showed that crop yields increased by an average of 5.0% when crop residue return was used, compared to no-straw treatment. The increases in yields attributed to crop residue return were affected little by mean annual temperature and soil total N content. The highest increases in yield occurred for cases with rainfall ≥ 800 mm (9.5%), ploughing depths of ≥ 20 cm (6.5%), corn crops (8.0%), crops with a full year of ripening (10.1%), no irrigation (11.9%), NK fertilizer (20.0%), low rates of N application (0–100 kg N ha^-1^; 10.8%), organic matter contents ≥ 15 g kg^-1^ (9.4%), effective nitrogen contents ≥100 mg kg^-1^ (10.3%), and pH ≤ 6.5 (11.2%). Moreover, our results suggest that crop residue return might increase crop yields and WUE most effectively where one or more of the following factors are present: corn crops, depth of tillage ≥ 20 cm, medium rates of N fertilization (0–150 kg ha^-1^), growth of one crop per year, high soil organic matter (≥ 15 g kg^-1^), and cold conditions (mean annual average temperature < 10 °C). Therefore, the effect of crop residue return on crop yield is related to climatic conditions, fertilization management, crop types, and soil properties. The optimal N amount, crop type, tillage type, and cropping system type for crop residue return were identified. Given the importance of global food security, greater attention should be paid to the optimization of crop residue return methods based on the factors identified in this study.

## Supporting information

S1 Data source (DOCX)

S1 Table Data information. (XLSX)

S2 Table Sensitivity analysis of crop residue return on crop yield. (DOCX)

S3 Table Categorical variables (Var), total number of paired observations of water use efficiency for straw return and no-straw treatments (k), specific levels of each Var (L), between-group heterogeneity (Qb), and significant P values produced by the meta-analysis. (DOCX)
