# Supplementary material for: A meta-analysis of the effects of crop residue return on crop yields and water use efficiency
Source: PLoS One. 2020 Apr 27;15(4):e0231740. doi: 10.1371/journal.pone.0231740 (PMC7185903; doi:10.1371/journal.pone.0231740)
Supplement: S1 Data source — (DOCX) [file pone.0231740.s001.docx]

**References**

Akhtar, K., Wang, W.Y., Khan, A., Ren, G.X., Afridi, M.Z., Feng, Y.Z., Yang, G.H. Wheat straw mulching with fertilizer nitrogen: an approach for improving soil water storage and maize crop productivity. Plant Soil Environment, 2018, 64(7): 330-337.

Chen, Y.L., Liu, T., Tian, X.H., Wang, X.F., Li, M., Wang, S.X., Wang, Z.H. Effects of plastic film combined with straw mulch on grain yield and water use efficiency of winter wheat in Loess Plateau. Field Crops Research, 2015, 172: 53–58.

Dong, Q.G., Yang, Y.C., Yu, K., Feng, H. Effects of straw mulching and plastic film mulching on improving soil organic carbon and nitrogen fractions, crop yield and water use efficiency in the Loess Plateau, China. Agricultural Water Management, 2018, 201: 133–143.

Fan, M.S., Liu, X.J., Jiang, R.F., Zhang, F.S., Lu, S.H., Zeng, X.Z., Christie, P. Crop yields, internal nutrient efficiency, and changes in soil properties in rice–wheat rotations under non-flooded mulching cultivation. Plant and Soil, 2005, 277: 265–276.

Fang, Q.L., Lin, Q., Liu, Y.G., Jiang, W., Li, L.Y., Zhao, C.X. Effects of conservation tillage on diurnal variation of photosynthesis in grain filling stage and yield of winter wheat. Journal of Agriculture, 2012,2(12):6-11. (In Chinese with English abstract)

Grahmann, K., Verhulst, N., Peña, R.J., Buerkert, A., Vargas-Rojas, L., Govaerts, B. Durum wheat (Triticum durum L.) quality and yield as affected by tillage–straw management and nitrogen fertilization practice under furrow-irrigated conditions. Field Crops Research, 2014, 164: 166–177.

He, Q.Y., Lv, W.G., Zheng, X.Q., Li, S.X., Zhang, J.Q., Wang, J.Q., Yuan, D.W., Zhang, H.L. Effects of different mechanical tillage methods on soil physical and chemical properties and rice yield with straw returning. Acta Agriculturae Shanghai, 2015, 31(5): 66-69. (In Chinese with English abstract)

Huang, T.M., Zheng, X.F., Hou, Y.Y., Li, X., Wang, Z.H. Yield and N， P and K uptake and utilization of winter wheat affected by straw return to soil. Journal of Plant Nutrition and Fertilizer, 2015, 21(4): 853-863. (In Chinese with English abstract)

Ji, Q., Sun, H.Y., Taraqqi, A.K., Wang, X.D. Impact of different tillage practices on soil organic carbon and water use efficiency under continuous wheat-maize binary cropping system．Chinese Journal of Applied Ecology，2014，25(4) : 1029 － 1035. (In Chinese with English abstract)

Kong, F.L., Zhang, H.L., Zhai, Y.L., Yuan, J.C., Chen, F. Effects of tillage methods on crop yield and water use characteristics in winter-wheat/summer-maize rotation system in the North China Plain. Chinese Journal of Eco-Agriculture, 2014, 22(7): 749−756. (In Chinese with English abstract)

Li, C.Z., Zhang, T.T., Feng, Y.Z., Ren, G.X., Yang, G.H. Effects of different rotation modes and straw returning on soil CO_2_ emission fluxes and moisture, heat, carbon and nitrogen conditions in wheat field. Agricultural Research in the Arid Areas, 2013, 31(6): 190-197. (In Chinese with English abstract)

Li, L.J., Wu, P.P., Geng, Y.A., Yao, W.Q., Wang, J.J. Effect of wheat straw addition with nitrogen application on physical-chemical properties of white paddy soil. Journal of Plant Nutrition and Fertilizer, 2016, 22(5): 1259–1266. (In Chinese with English abstract)

Li, L.L., Huang, G.B., Qin S.H., Yu A.Z. Effect of Conservation Tillage on Dry Matter Accumulating and Yield of Winter Wheat in Oasis Area. Acta Agronomica Sinica, 2011, 37(3): 514−520. (In Chinese with English abstract)

Li, W., Qiao, Y.Q., Chen, H., Cao, C.F., Du, S.Z., Zhao, Z. Effects of combined straw and N application on the physicochemical properties of lime concretion black soil and crop yields． Acta Ecologica Sinica，2014, 34( 17): 5052-5061．(In Chinese with English abstract)

Li, Z., Lai, X.F., Yang, Q., Yang, X., Cui, S., Shen, Y.Y. In search of long-term sustainable tillage and straw mulching practices for a maize-winter wheat-soybean rotation system in the Loess Plateau of China. Field Crops Research, 2018, 217: 199–210.

Lu, W.T., Jia, Z.K., Gao, F., Li, Y.P., Hou, X.Q. Effects of Straw Returning on Soil Water and Crop Productivity in the Rainfed Area of Southern Ningxia, China. Journal of Agro-Environment Science, 2011,30(1):93-99. (In Chinese with English abstract)

Ma X.L., Jia, Z.K., Xiao, E.S., Wang, W.Y., Liu, T., Liu, Y.H., Cui, R.M. Effects of wheat-residue application on soil water and water use efficiency in the Weibei Loess Plateau. Agricultural Research in the Arid Areas, 2010, 28(5): 59-64. (In Chinese with English abstract)

Mu, P., Zhang, E.H., Wang, H.N., Fang, Y.F. Effects of continuous straw return to soil on maize growth and soil chemical and physical characteristics. Chinese Journal of Eco-Agriculture, Mar. 2012, 20(3): 291-296. (In Chinese with English abstract)

Pang, H.C., Li, Y.Y., Yang, J.S., Liang, Y.S. Effect of brackish water irrigation and straw mulching on soil salinity and crop yields under monsoonal climatic conditions. Agricultural Water Management, 2010, 97: 1971–1977.

Peng, W.D., Chu, C.X., Zhong, Y.Q., Lai, W.H., Zhang, H.B., Huang, L.Y., Shi, X.X., Wei, J.L. Effects of returning to field of sweet corn straw on soil fertility, yield and benefit. Guangdong Agricultural Sciences, 2015.15.009. (In Chinese with English abstract)

Stagnari, F., Galieni, A., Speca, S., Cafiero, G., Pisante, M. Effects of straw mulch on growth and yield of durum wheat during transition to Conservation Agriculture in Mediterranean environment. Field Crops Research, 2014, 167: 51–63.

Su, W., Lu, J.W., Wang, W.N., Li, X.K., Ren, T., Cong, R.H. Influence of rice straw mulching on seed yield and nitrogen use efficiency of winter oilseed rape (Brassica napus L.) in intensive rice–oilseed rape cropping system. Field Crops Research, 2014, 159: 53–61.

Tan, S., Wang, Q.J., Xu, D., Zhang, J.H., Shan, Y.Y. Evaluating effects of four controlling methods in bare strips on soil temperature, water, and salt accumulation under film-mulched drip irrigation. Field Crops Research, 2017, 214: 350–358.

Tong, X.X., Jiang, W. Effects of tillage managements in wheat-maize whole season on grain filling characteristics and yield of summer maize. Acta Agronomica Sinica, 2014, 29 (2): 141 -145. (In Chinese with English abstract)

Wang, F.S., Chang, L., Sun, Z.H., Huang, C.X., Han, F.X., Chai, S.X. Effects of straw returning and plastic film mulching on dryland maize yield in Northwest China. Journal of irrigation and drainage, 2014, 33(6): 109-112. (in Chinese with English abstract)

Wang, J.D., Zhang, Y.Q., Gong, S.H., Xu, D., Juan, S., Zhao, Y.F. Evapotranspiration, crop coefficient and yield for drip-irrigated winter wheat with straw mulching in North China Plain. Field Crops Research, 2018, 217: 218–228.

Wang, N., Liu, Y.G., Zhang, H.S., Li, L.Y., Lin, Q. Effects of coupling of precise straw-return and nitrogen fertilizer on photosynthetic characteristics after anthesis and yield of winter wheat. Acta Agriculturae Boreali-Sinica, 2012, 27(6): 185-190. (In Chinese with English abstract)

Wang, N.N., Zhu, F.L., Zhang, C.F., Jia, H.B., Zhu, B.G., Meng, Q.Y., Song, Y.B., Li, Y. Effect of different returning method of straw to soil on soil chemical property and soybean yield, Soil and Fertilizer Sciences in China, 2016(1). (In Chinese with English abstract)

Wang, Z.Y., Bai, Y.L., Yang, L.P., Lu, Y.L., Wang, L., Wang, H. Effects of application of potassium fertilizer and straw returning on crop yields and soil potassium balance in low- yielding fields. Plant Nutrition and Fertilizer Science, 2012，18( 4) : 900-906. (In Chinese with English abstract)

Wu, X.S., Zhou, X.L., Cao, F.M., Zhu, B.C., Zhao, T.K., Shen, D.L. Effects of different fertilization on the corn yield and soil enzyme activity in corn growth period. Soil and Fertilizer Sciences in China, 2015 (1). (In Chinese with English abstract)

Yan, C., Yan, S.S., Wang, J.R, Dong, S.K., Gong, Z.P. Effect of rice straw retention and potassium fertilizer application in cold region on soluble potassium content in the soil solution and rice yield[J]. Journal of Northeast Agricultural University, 2015, 46(5): 16-21. (in Chinese with English abstract)

Yan, Q.Y., Yang, F., Dong, F., Lu, J.X., Li, F., Duan, Z.Q., Zhang, J.C., Lou, G. Yield loss compensation effect and water use efficiency of winter wheat under double-blank row mulching and limited irrigation in northern China. Field Crops Research, 2018, 216: 63–74.

Yuan, L., Zhang, X., Yang, J., Yang, C.L., Cao, X.C., Wu L.H. Effects of different cultivation methods and straw incorporation on grain yield and nutrition quality of rice. Acta Agronomica Sinica, 2013, 39(2): 350-359. (In Chinese with English abstract)

Zhan, X.M., Peng, J., Li, X.L., Li, T.T., Han, X.R., Song, T., Pan, Q.L. Effects of tillage and crop residues incorporation on spring maize yield and physical and chemical properties of soil. Acta Agriculturae Boreali-Sinica, 2014, 29(3): 204 -209. (In Chinese with English abstract)

Zhang, L., Zheng, J.C., Chen, L.G., Shen, M.X., Zhang, X., Zhang, M.Q., Bian, X.M., Zhang, J., Zhang, W.J. Integrative effects of soil tillage and straw management on crop yields and greenhouse gas emissions in a rice–wheat cropping system. European Journal of Agronomy, 2015, 63: 47–54.

Zhang, Q., Zhang, L., Jiao, N.Y., Li, C.H., Fu, G.Z. Effects of the alternate years deep plowing on leaf senescence after anthesis and grain yield of summer maize. Journal of Maize Sciences, 2013, 21(5)：62-65. (In Chinese with English abstract)

Zhang, S.S., Cai, L.Q., Zhang, R.Z. Effect of tillage measures on the soil potassium contents and yields in the double sequence rotation system. Journal of Gansu Agricultural University, 2012, (3): 32-38. (In Chinese with English abstract)

Zhang, Y.Y., Han, H.K., Zhang, D.Z., Li, J., Gong, X.W., Baili Feng, B.L., Xue, Z.H., Yang, P. Effects of ridging and mulching combined practices on proso millet growth and yield in semi-arid regions of China. Field Crops Research, 2017, 213: 65–74.

Zhao, Y.L., Guo, H.B., Xue, Z.W., Mu, X.Y., Li, C.H. Effects of tillage and straw returning on biomass and water use efficiency in a winter wheat and summer maize rotation system. Acta Agronomica Sinica, 2014, 40(10): 1797−1807. (In Chinese with English abstract)
