# Supplementary material for: A meta-analysis of the effects of crop residue return on crop yields and water use efficiency
Source: PLoS One. 2020 Apr 27;15(4):e0231740. doi: 10.1371/journal.pone.0231740 (PMC7185903; doi:10.1371/journal.pone.0231740)
Supplement: S2 Table — (DOCX) [file pone.0231740.s003.docx]

**Table S2.** Sensitivity analysis of crop residue return on crop yield

| No | The change in  crop yield (%) |  | 95% CI (%) | |  | No | The change in  crop yield (%) |  | 95% CI (%) | |
| --- | --- | --- | --- | --- | --- | --- | --- | --- | --- | --- |
|  |  |  | Lower limit | Upper limit |  |  |  |  | Lower limit | Upper limit |
| 1 | 4.50 |  | 3.76 | 5.23 |  | 78 | 4.52 |  | 3.79 | 5.26 |
| 2 | 4.48 |  | 3.75 | 5.22 |  | 79 | 4.52 |  | 3.79 | 5.26 |
| 3 | 3.77 |  | 3.42 | 5.90 |  | 80 | 4.51 |  | 3.78 | 5.25 |
| 4 | 4.48 |  | 3.37 | 5.87 |  | 81 | 4.51 |  | 3.77 | 5.25 |
| 5 | 4.44 |  | 3.71 | 5.18 |  | 82 | 4.50 |  | 3.76 | 5.24 |
| 6 | 4.43 |  | 3.69 | 5.17 |  | 83 | 4.50 |  | 3.76 | 5.24 |
| 7 | 4.49 |  | 3.76 | 5.23 |  | 84 | 4.49 |  | 3.76 | 5.23 |
| 8 | 4.71 |  | 3.92 | 5.50 |  | 85 | 4.49 |  | 3.76 | 5.23 |
| 9 | 4.49 |  | 3.76 | 5.23 |  | 86 | 4.49 |  | 3.76 | 5.23 |
| 10 | 4.50 |  | 3.76 | 5.23 |  | 87 | 4.49 |  | 3.76 | 5.23 |
| 11 | 4.47 |  | 3.74 | 5.21 |  | 88 | 4.59 |  | 3.86 | 5.33 |
| 12 | 4.46 |  | 3.73 | 5.20 |  | 89 | 4.50 |  | 3.76 | 5.24 |
| 13 | 4.45 |  | 3.69 | 5.21 |  | 90 | 4.49 |  | 3.75 | 5.23 |
| 14 | 4.65 |  | 3.89 | 5.38 |  | 91 | 4.49 |  | 3.75 | 5.23 |
| 15 | 4.44 |  | 3.70 | 5.18 |  | 92 | 4.48 |  | 3.75 | 5.22 |
| 16 | 4.39 |  | 3.65 | 5.13 |  | 93 | 4.48 |  | 3.75 | 5.22 |
| 17 | 4.56 |  | 3.82 | 5.3 |  | 94 | 4.47 |  | 3.74 | 5.21 |
| 18 | 4.47 |  | 3.74 | 5.21 |  | 95 | 4.47 |  | 3.73 | 5.21 |
| 19 | 4.54 |  | 3.81 | 5.29 |  | 96 | 4.50 |  | 3.76 | 5.24 |
| 20 | 4.47 |  | 3.73 | 5.21 |  | 97 | 4.48 |  | 3.75 | 5.22 |
| 21 | 4.42 |  | 3.68 | 5.16 |  | 98 | 4.56 |  | 3.83 | 5.3 |
| 22 | 4.64 |  | 3.90 | 5.38 |  | 99 | 4.55 |  | 3.82 | 5.29 |
| 23 | 4.51 |  | 3.77 | 5.25 |  | 100 | 4.54 |  | 3.8 | 5.28 |
| 24 | 4.49 |  | 3.76 | 5.23 |  | 101 | 4.54 |  | 3.8 | 5.28 |
| 25 | 4.48 |  | 3.75 | 5.22 |  | 102 | 4.53 |  | 3.79 | 5.27 |
| 26 | 4.47 |  | 3.74 | 5.21 |  | 103 | 4.53 |  | 3.79 | 5.27 |
| 27 | 4.47 |  | 3.73 | 5.21 |  | 104 | 4.52 |  | 3.79 | 5.26 |
| 28 | 4.46 |  | 3.73 | 5.20 |  | 105 | 4.52 |  | 3.79 | 5.26 |
| 29 | 4.46 |  | 3.73 | 5.19 |  | 106 | 4.52 |  | 3.78 | 5.26 |
| 30 | 4.50 |  | 3.76 | 5.24 |  | 107 | 4.51 |  | 3.78 | 5.25 |
| 31 | 4.49 |  | 3.75 | 5.23 |  | 108 | 4.51 |  | 3.77 | 5.25 |
| 32 | 4.46 |  | 3.73 | 5.20 |  | 109 | 4.50 |  | 3.77 | 5.24 |
| 33 | 4.44 |  | 3.71 | 5.18 |  | 110 | 4.55 |  | 3.82 | 5.29 |
| 34 | 4.45 |  | 3.71 | 5.19 |  | 111 | 4.55 |  | 3.82 | 5.29 |
| 35 | 4.43 |  | 3.69 | 5.17 |  | 112 | 4.50 |  | 3.76 | 5.24 |
| 36 | 4.43 |  | 3.69 | 5.17 |  | 113 | 4.47 |  | 3.73 | 5.21 |
| 37 | 4.42 |  | 3.69 | 5.16 |  | 114 | 4.40 |  | 3.66 | 5.14 |
| 38 | 4.49 |  | 3.76 | 5.23 |  | 115 | 4.37 |  | 3.63 | 5.11 |
| 39 | 4.49 |  | 3.76 | 5.23 |  | 116 | 4.49 |  | 3.76 | 5.23 |
| 40 | 4.49 |  | 3.76 | 5.23 |  | 117 | 4.46 |  | 3.73 | 5.2 |
| 41 | 4.49 |  | 3.76 | 5.23 |  | 118 | 4.55 |  | 3.81 | 5.29 |
| 42 | 4.49 |  | 3.76 | 5.23 |  | 119 | 4.50 |  | 3.76 | 5.24 |
| 43 | 4.49 |  | 3.76 | 5.23 |  | 120 | 4.50 |  | 3.76 | 5.24 |
| 44 | 4.51 |  | 3.77 | 5.25 |  | 121 | 4.56 |  | 3.82 | 5.3 |
| 45 | 4.50 |  | 3.77 | 5.24 |  | 122 | 4.49 |  | 3.75 | 5.23 |
| 46 | 4.50 |  | 3.76 | 5.24 |  | 123 | 4.49 |  | 3.76 | 5.23 |
| 47 | 4.49 |  | 3.76 | 5.23 |  | 124 | 4.50 |  | 3.76 | 5.24 |
| 48 | 4.49 |  | 3.76 | 5.23 |  | 125 | 4.49 |  | 3.76 | 5.23 |
| 49 | 4.49 |  | 3.75 | 5.23 |  | 126 | 4.48 |  | 3.75 | 5.22 |
| 50 | 4.53 |  | 3.79 | 5.28 |  | 127 | 4.38 |  | 3.65 | 5.12 |
| 51 | 4.71 |  | 3.92 | 5.5 |  | 128 | 4.59 |  | 3.86 | 5.33 |
| 52 | 4.51 |  | 3.77 | 5.25 |  | 129 | 4.58 |  | 3.84 | 5.32 |
| 53 | 4.49 |  | 3.76 | 5.23 |  | 130 | 4.58 |  | 3.84 | 5.32 |
| 54 | 4.48 |  | 3.75 | 5.22 |  | 131 | 4.57 |  | 3.84 | 5.31 |
| 55 | 4.47 |  | 3.73 | 5.21 |  | 132 | 4.54 |  | 3.80 | 5.28 |
| 56 | 4.46 |  | 3.73 | 5.2 |  | 133 | 4.54 |  | 3.80 | 5.28 |
| 57 | 4.46 |  | 3.73 | 5.2 |  | 134 | 4.52 |  | 3.79 | 5.26 |
| 58 | 4.50 |  | 3.76 | 5.24 |  | 135 | 4.50 |  | 3.77 | 5.24 |
| 59 | 4.51 |  | 3.77 | 5.25 |  | 136 | 4.50 |  | 3.76 | 5.24 |
| 60 | 4.50 |  | 3.76 | 5.24 |  | 137 | 4.43 |  | 3.70 | 5.17 |
| 61 | 4.49 |  | 3.75 | 5.23 |  | 138 | 4.41 |  | 3.67 | 5.15 |
| 62 | 4.49 |  | 3.75 | 5.23 |  | 139 | 4.54 |  | 3.80 | 5.28 |
| 63 | 4.6 |  | 3.86 | 5.34 |  | 140 | 4.44 |  | 3.71 | 5.19 |
| 64 | 4.54 |  | 3.81 | 5.29 |  | 141 | 4.53 |  | 3.80 | 5.27 |
| 65 | 4.52 |  | 3.78 | 5.26 |  | 142 | 4.50 |  | 3.76 | 5.24 |
| 66 | 4.52 |  | 3.76 | 5.24 |  | 143 | 4.49 |  | 3.76 | 5.23 |
| 67 | 4.48 |  | 3.74 | 5.22 |  | 144 | 4.49 |  | 3.76 | 5.23 |
| 68 | 4.55 |  | 3.81 | 5.30 |  | 145 | 4.49 |  | 3.75 | 5.22 |
| 69 | 4.48 |  | 3.74 | 5.22 |  | 146 | 4.49 |  | 3.75 | 5.23 |
| 70 | 4.48 |  | 3.74 | 5.22 |  | 147 | 4.47 |  | 3.73 | 5.21 |
| 71 | 4.48 |  | 3.74 | 5.22 |  | 148 | 4.48 |  | 3.74 | 5.22 |
| 72 | 4.44 |  | 3.71 | 5.18 |  | 149 | 4.46 |  | 3.73 | 5.20 |
| 73 | 4.4 |  | 3.66 | 5.14 |  | 150 | 4.48 |  | 3.75 | 5.22 |
| 74 | 4.4 |  | 3.66 | 5.14 |  | 151 | 4.44 |  | 3.7 | 5.18 |
| 75 | 4.36 |  | 3.63 | 5.11 |  | 152 | 4.45 |  | 3.71 | 5.19 |
| 76 | 4.50 |  | 3.76 | 5.24 |  | 153 | 4.42 |  | 3.69 | 5.16 |
| 77 | 4.46 |  | 3.73 | 5.20 |  | 154 | 4.49 |  | 3.76 | 5.23 |
