# Supplementary material for: A meta-analysis of the effects of crop residue return on crop yields and water use efficiency
Source: PLoS One. 2020 Apr 27;15(4):e0231740. doi: 10.1371/journal.pone.0231740 (PMC7185903; doi:10.1371/journal.pone.0231740)
Supplement: S3 Table — (DOCX) [file pone.0231740.s004.docx]

**Table S3.** Categorical variables (Var), total number of paired observations of water use efficiency for straw return and no-straw treatments (k), specific levels of each Var (L), between-group heterogeneity (Qb), and significant P values produced by the meta-analysis.

| **No** | **Var.** | **k** | **L1** | **L2** | **L3** | **L4** | **Qb** | **p** |
| --- | --- | --- | --- | --- | --- | --- | --- | --- |
| 1 | Crop types | 47 | Millet | Soybean | Corn | Wheat | 9.6753 | 0.0248 |
| 2 | Tillage types | 34 | Rotary tillage | Chisel plow tillage | No tillage | Mouldboard ploughing tillage | 1.8917 | 0.5282 |
| 3 | Fertilizer types | 49 | N | NP+organic fertilizer | NPK | NP | 1.78 | 0.5926 |
| 4 | Experimental duration (year) | 49 | ≤2 | 3-10 |  |  | 1.3508 | 0.2230 |
| 5 | Depth of tillage (cm) | 27 | <20 | ≥20 |  |  | 0.8715 | 0.3384 |
| 6 | Amount of N fertilizer (kg ha^-1^) | 39 | >150 | 100-150 | 0-100 |  | 11.7035 | 0.0066 |
| 7 | Irrigation condition | 40 | No irrigation | Irrigation |  |  | 4.7605 | 0.0136 |
| 8 | Cropping systems | 48 | One crop a year | Two crops a year |  |  | 12.0566 | 0.0028 |
| 9 | Soil organic matter (g kg^-1^) | 32 | ≥15 | 10-15 | ≤10 |  | 10.7167 | 0.0094 |
| 11 | Mean annual temperature (^o^C) | 29 | 10-15 | <10 |  |  | 10.3673 | 0.0018 |
